# Interprofessional survey on communication skills in veterinary and veterinary-related education in Germany

**DOI:** 10.1186/s12909-021-02938-8

**Published:** 2021-09-29

**Authors:** Michèle Rauch, Sandra Wissing, Andrea Tipold, Christin Kleinsorgen

**Affiliations:** 1 Centre for E-Learning, Didactics and Educational Research (ZELDA), Clinical Skills Lab, University of Veterinary Medicine Hannover, Foundation, Bischofsholer Damm 15, 30171 Hannover, Germany; 2 Small Animal Clinic, University of Veterinary Medicine Hannover, Foundation, Bünteweg 9, 30559 Hannover, Germany; 3 Centre for E-Learning, Didactics and Educational Research (ZELDA), E-Learning-Department, University of Veterinary Medicine Hannover, Foundation, Bünteweg 2, 30559 Hannover, Germany

**Keywords:** Communication skills, Interprofessional education, Veterinary and veterinary-related professions, Professional skills training

## Abstract

**Background:**

Communication is an indispensable skill in the everyday working life of a veterinary team. In German higher educational institutions, communication skills training is explicitly mentioned in the curricula of veterinary assistants, including veterinary nurses and veterinary technicians, and of animal keepers, but not for undergraduate veterinary medicine. Little is known about interprofessional education in veterinary medicine and related professions. Therefore, the purpose of this study is to describe and explore the current interprofessional state of knowledge regarding communication skills of the aforementioned groups in Germany.

**Methods:**

To explore the perception, assess the knowledge and identify the attitude regarding communication skills and interprofessional training, an online survey was distributed. The survey was sent to all five veterinary higher educational institutions, 38 schools for veterinary assistants and 15 schools for animal keepers throughout Germany.

**Results:**

In total, 294 veterinary students, 111 veterinary assistant trainees and 62 animal keeper trainees participated. The majority of participants (98.07%, *n* = 458) perceived communication skills as highly important for their everyday work. In total, 413 participants (88.44%) felt that their communication skills needed improvement and more than half admitted having difficulties in effective communication (59.31%; *n* = 277). In addition, 62.74% of respondents (*n* = 293) were not sufficiently informed about the training content of their future colleagues. Most were convinced that training could positively influence on their communication with clients (95.72%; *n* = 447) and the team (92.29%; *n* = 431), and 76.45% of respondents (*n* = 357) wished to participate in an interprofessional training.

**Conclusions:**

Results of this study confirm that communication skills are perceived as highly important for professional life. Students and trainees show a great interest in communication skills and interprofessional training. The findings indicate that appropriate adjustments to existing curricula are necessary in Germany.

## Background

Communication is one of the most frequently required and therefore indispensable skill in the daily work of a veterinary professional [1]. A kind, gentle, respectful and informative interaction can be identified as the main factor when pet owners are asked how they choose their veterinarian [2]. Communication varies due to different contexts and contents and represents a core competence of a veterinarian besides medical knowledge and medical examination skills [3]. For this reason, effective communication skills play a major role for veterinarians and the veterinary team and are fundamental for the success of a veterinary practice [4].

Due to the importance of the human-animal bond and the associated public demand for extended services and high-quality care in veterinary medicine [2], the requirements for members of the veterinary team are increasing [4]. An increasing demand for veterinary specialists can be observed and veterinary teams collaborating in cooperate veterinary group practices or veterinary clinics are growing in size [5]. This collaboration is defined as interprofessional collaborative practice [6] and includes, alongside other skills, interprofessional communication as a core competence [7]. Due to the high relevance of communication skills in the veterinary field when working with clients and members of the team, the focus in this paper is on interprofessional communication. Although the veterinary team may include various professions (practice managers, physiotherapists, nutritionists etc. [4]), this study focuses only on veterinary students, veterinary assistant trainees and animal keeper trainees. The professions of veterinary assistants and animal keepers are nationally recognised and certified professions with a three-year training period in Germany [8]. Veterinary assistants assist veterinarians during the examination, in the treatment and care of animals, in advising animal owners and carry out organisational and administrative work. Animal keepers can also be employed in a veterinary practice, where they are responsible for the animal care and assisting with procedures and treatments [9]. Similar to veterinarians, both professions come in close contact with clients in their everyday professional life.

When veterinarians or other members of the team fail to communicate effectively, this can quickly lead to misunderstandings or treatment errors and give cause for complaint [10, 11]. Thus, misconceptions or a lack of knowledge about the role of team members from other professions can quickly result in conflicts [12]. Difficulties can arise both in the hierarchical structure and in internal communication [4]. A pronounced hierarchy gap or a lack of interprofessional communication skills among team members can lead to stereotypical thinking patterns, feelings of oppression, rivalry and dissatisfaction [4]. Consequently, a successful, interprofessional cooperation can no longer be guaranteed. To be able to deal with such situations, effective interprofessional communication skills, with all team members being aware of their own fields of responsibility as well as those of their colleagues from other professions and understanding and respect of the distribution of roles are therefore of paramount importance [4].

A potential means to address this issue and to guarantee effective interprofessional cooperation in the future is interprofessional education [4, 12]. The World Health Organization defines interprofessional education as taking place when “students from two or more professions learn about, from and with each other to enable effective collaboration and improve health outcomes” [6]. Interprofessional education successfully enables effective interprofessional collaboration and demonstrably promotes students’ awareness of the importance of other health professions and their shared responsibilities [12]. In this way, professional isolation and hierarchical views can be reduced, the understanding of mutual tasks and common goals can be strengthened and both interprofessional communication and teamwork can be improved [13].

Adequate veterinary education, which specifically includes the promotion of communication skills, is therefore essential [14, 15]. In German veterinary higher educational institutions, the training of communication skills is not explicitly named in the curriculum of undergraduate veterinary medicine studies, but rather listed as an implicit teaching goal or within the hidden curriculum [16]. In contrast, communication with clients and within the team is an integral part of the training of veterinary assistants and animal keepers in Germany, with explicit assessment for the veterinary assistants and specific learning objectives for animal keepers [8, 9].

Little is known about interprofessional education related to the veterinary team [13] and in veterinary training in German speaking countries [17]. Interprofessional education programmes to promote interprofessional cooperation are currently rare for all health professions [17] and despite the close cooperation between veterinarians and veterinary assistants, little emphasis has been placed on interprofessional education in veterinary training so far [13]. However, interprofessional education in veterinary medicine is gaining attention and can draw on insights from human medicine [18].

The aim of this study is to record the current interprofessional state of knowledge regarding communication skills of veterinary undergraduate students, veterinary assistant trainees and animal keeper trainees in Germany. The study specifically focusses on the objectives to explore participants’ perception of the value of communication skills in the veterinary workplace, to self-assess participants’ own communication skills and to identify their attitude towards communication teaching and interprofessional education. The results should enable the implementation of an adequate interprofessional communication skills training and provide a starting point for a common training basis in the curricula or early postgraduate phase.

## Methods

### Survey

An online survey was created and distributed nationally in the period from 11 May 2020 to 21 July 2020. The survey was sent to all five national veterinary higher educational institutions (with approximately 6300 undergraduate students), to 38 schools for veterinary assistants and to 15 schools for animal keepers nationwide. All semesters and all years of training were included, as communication training occurs during all stages of the education. Not all schools offering training for veterinary assistants and animal keepers could be contacted, as a complete list of all schools or the current number of trainees is not available. The link to the survey was sent all across the nation by e-mail via the semester distribution lists of the veterinary educational institutions, various internet portals and by contacting the schools and educational institutions directly.

The survey was designed with the online software LimeSurvey®, comprising a total of nine pages with eight sections and containing 85 items, whereby a pre-defined logic allowed between 82 and 85 questions to be answered. Only an excerpt of relevant questions is presented in this article. In addition to providing personal data, the participants were asked to answer a multiple-choice test to assess theoretical communication content and a section of questions to identify the value of communication in the veterinary practice, to rate their own communication skills with clients and within the veterinary team and their demands for and interest in communication training and interprofessional education. Furthermore, participants were able to give free text answers about situations which they find difficult or easy when communicating with clients or within the veterinary team. Free text answers were collected by means of qualitative content analysis in accordance with Mayring [19]. Within the framework of a structured content analysis, the text material was coded, assigned to predefined categories and then interpreted. The survey contained single- and multiple-choice questions, rating questions with four-point Likert items and free answer questions. The knowledge test consisted of ten single best answer questions. In addition, the answer option “I don’t know” was offered. The development of the survey was based on literature research about veterinary communication and on existing questionnaires including the “Communication Skills Attitude Scale” (CSAS) [20] and the study of Meehan and Menniti [21]. Furthermore, questions relating to research of interprofessional needs assessment were added and validated within the working group.

The Data Protection Officer reviewed the proposed project regarding observance of the Data Protection Law and gave permission to perform the study. All experiments were performed in accordance with relevant guidelines and regulations and informed consent was obtained from all subjects involved in the study. The Ethics Committee or the Thesis Commission of the University of Veterinary Medicine Hannover, Foundation, Hannover, Germany approved the conception, method and publication of the study. All obtained data were evaluated in compliance with the EU’s General Data Protection Regulation.

### Statistical analysis

The descriptive and statistical analysis was carried out using Microsoft® Office Excel 2016 and SAS® Enterprise Guide 7.1. The Chi-squared test was chosen to test whether there was an association between education, gender, age or previous professional training of the participants and their answers to the survey. For some questions, Fisher’s exact test was used due to the high number of values with expected frequencies below five. The significance level for both tests was 5%. In order to obtain valid test results, the following answer options were combined: “Very good” and “Good” were summarised to “Good”; “Poor” and “Very poor” were combined to “Poor”. “Fully applicable” / “More likely to apply” and “Yes, definitely” / “Yes, rather” were combined to “Agree”; “Rather not applicable” / “Not applicable at all” and “No, rather not” / “No, not at all” were summarised to “Disagree”. For the comparison of the overall results between the different professional groups in the multiple-choice test, an analysis of variance for independent factors (one-way ANOVA) was carried out.

## Results

### Demographics

**Fig. 1:**
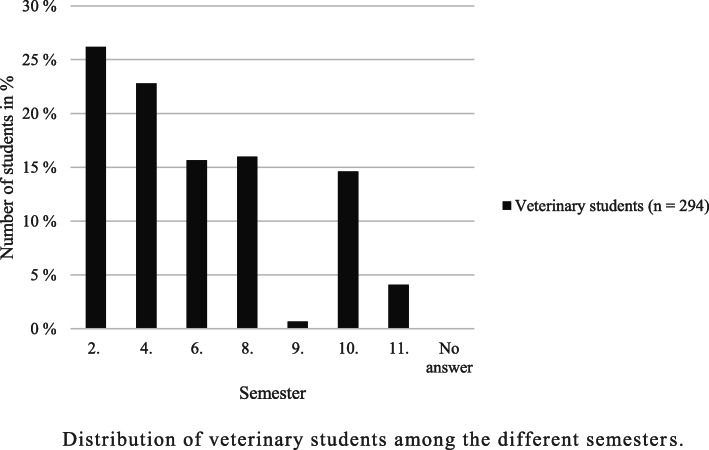
Distribution of veterinary students among the different semesters. (In German veterinary higher educational institutions, odd semesters take place during the winter and even semesters during the summer. Since the survey was conducted during the summer, 0 participants were in semesters 1, 3, 5 and 7 at that time. Due to sabbatical years or other reasons, students may alternate between even and odd semesters)

**Fig. 2:**
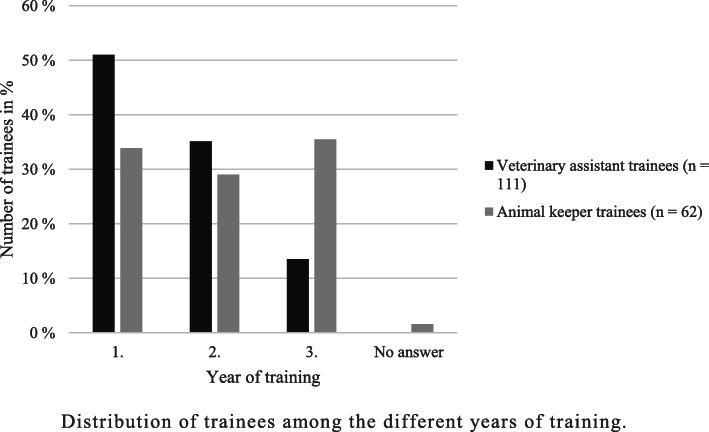
Distribution of trainees among the different years of training

A total of 689 surveys were filled in. Of these, 467 surveys were answered completely at least up to the rating questions and were used for the following evaluation. As previously mentioned, the population size was not detectable for veterinary trainees due to a lack of data, whereas approximately 5% of German veterinary undergraduate students participated in this national survey. Veterinary students were the largest proportion of participants (62.96%; *n* = 294), 23.77% (*n* = 111) of respondents were veterinary assistant trainees and 13.28% (*n* = 62) were animal keeper trainees. The majority of participants in the survey were female (89.94%; *n* = 420), 8.14% (*n* = 38) were male and 0.64% (*n* = 3) selected the answer option “Other” when asked about their gender. A total of 1.28% (*n* = 6) did not indicate their gender. The high proportion of female participants could be noted for each of the three surveyed groups (veterinary undergraduate students: 91.84%; *n* = 270, veterinary assistant trainees: 95.50%; *n* = 106, animal keeper trainees: 70.97%; *n* = 44). The participants ranged in age from 17 to 40 years, with an average age of 22.88 years. A total of 308 participants (65.95%) stated that they were in their first training programme at the time of the survey. A total of 10.49% (*n* = 49) of the respondents had already started, and 22.91% (*n* = 107) had already completed another training programme. The distribution of veterinary students among the different semesters and of trainees among the different years of training is shown in Figs. 1 and 2.

### Participants’ perception of the value of communication skills in the veterinary workplace

Even though the absolute majority of participants agreed with the importance of communication skills (98.07%; *n* = 458) (Table 1), it was found that animal keeper trainees denied significantly more often (9.68%) that they perceived communication skills as important for their professional everyday life (Fisher’s test: *p* < 0.0001). Furthermore, male participants denied significantly more often (10.53%) that communication skills were important for their everyday professional life compared to female participants (0.71%) (Fisher’s test: *p* = 0.0011). Most of the participants (77.30%; *n* = 361) agreed that communication skills were equally important as clinical knowledge. A significantly lower proportion of animal keeper trainees (62.90%) than of veterinary students and veterinary assistant trainees (79.25 and 80.18%) agreed that communication skills were as important as clinical knowledge (χ^2^: *p* = 0.0098). Many respondents (73.02%; *n* = 341) had already experienced conflict situations in a professional context that had been triggered by a lack of communication skills.

**Table 1:** Participants’ perception of the value of communication skills in the veterinary workplace

Items	Veterinary students (*n* = 294)		Trainees for veterinary nurses (*n* = 111)		Animal keeper trainees (*n* = 62)		All participants (*n* = 467)		
	1	2	1	2	1	2	1	2	No answer
Communication skills are important for my everyday work.	99.32	0.00	99.10	0.90	90.32	9.68	98.07	1.50	0.43
Communication is helpful in carrying out euthanasia.	96.60	3.40	96.40	3.60	91.94	8.06	95.93	4.07	0.00
Developing communication skills is as important as expanding clinical knowledge.	79.25	20.07	80.18	18.92	62.90	37.10	77.30	22.06	0.64
I have already experienced conflict situations in a professional context that were triggered by a lack of communication skills.	67.69	32.31	82.88	17.12	80.65	19.35	73.02	26.98	0.00

Participants’ rating of the value of communication skills in the veterinary workplace are presented, with 1 = Agree and 2 = Disagree. Values are stated in percent (%). For each subgroup of the professions, the data of “No answer” are not displayed, but can be calculated as missing difference to 100%

### Participants’ assessment of their own communication skills

The multiple-choice test, assessing participants’ theoretical communication skills, consisted of basic questions on communication theory. Respondents selected the right answer for an average of 60.32% of the questions. Veterinary students answered an average of 62.31%, veterinary assistant trainees 57.85% and animal keeper trainees 55.48% of the questions correctly. A significant difference in the correct answers to the questions by the different professional groups could be found (*p* = 0.0109). Thus, veterinary students chose the correct answer significantly more often compared to animal keeper trainees. No significant difference could be found between the correct answers of veterinary students and veterinary assistant trainees or between veterinary assistant trainees and animal keeper trainees (*p* = 0.1640; *p* = 0.1607). The term of interprofessional communication was familiar to 38.12% (*n* = 178) of participants. A significantly higher proportion of veterinary students (46.94%) and animal keeper trainees (38.71%) than of veterinary assistant trainees (14.41%) were likely to know what was meant by the term of interprofessional communication (χ^2^: *p* < 0.0001).

Regarding participants’ self-assessment of their own communication skills (Table 2), no statistically significant difference between the professional groups with regard to their self-assessment could be demonstrated (χ^2^: *p* = 0.5893). Furthermore, participants from higher age groups or with previous professional training did not rate their own communication skills significantly better than those from lower age groups or without previous professional training. Even though most of the participants (89.29%; *n* = 417) assessed their own communication skills as good or very good, 413 people (88.44%) felt that their communication skills needed to be improved. Veterinary students identified their communication skills significantly more frequent as being in need of improvement (93.20%) than veterinary assistant trainees (81.98%) and animal keeper trainees (77.42%) (χ^2^: *p* = 0.0001). Likewise, veterinary students agreed significantly more often (65.31%) that they had problems with communication than veterinary assistant trainees (54.95%) or animal keeper trainees (38.71%) (χ^2^: *p* = 0,0003). Participants from higher age groups as well as those with previous professional training were equally likely to state that their communication skills needed to be improved than those from the lower age groups or those without previous professional training. In contrast, a significantly lower proportion of respondents with previous professional training stated that they had problems when communicating in their everyday working life (48.60%) than those who were currently in their first training or studies (63.64%) (χ^2^: *p* = 0.0226).

**Table 2:** Participants’ self-assessment of their own communication skills

Items	Veterinary students (*n* = 294)		Trainees for veterinary nurses (*n* = 111)		Animal keeper trainees (*n* = 62)		All participants (*n* = 467)		
	1	2	1	2	1	2	1	2	No answer
How would you rate your own communication skills?	88.44	11.56	91.89	8.11	88.71	9.68	89.29	10.49	0.21
I think that I can use appropriate eye contact.	81.29	18.37	88.29	11.71	83.87	16.13	83.30	16.49	0.21
I think that I can use the tone of my voice in a controlled way during counselling.	78.91	20.75	89.19	10.81	80.65	19.35	81.58	18.20	0.21
I believe that I can actively listen to a client in a conversation.	96.60	3.06	95.50	3.60	93.55	6.45	95.93	3.64	0.43
I let my counterpart speak out in a conversation.	93.54	6.12	98.20	1.80	98.39	1.61	95.29	4.50	0.21
I consciously make sure to control my facial expression in a conversation.	63.27	36.39	78.38	21.62	67.74	32.26	67.45	32.33	0.21
I consciously pay attention to use non-verbal communication appropriately.	51.70	47.96	68.47	31.53	51.61	48.39	55.67	44.11	0.21
Do you think your communication skills should be improved?	93.20	6.80	81.98	18.02	77.42	22.58	88.44	11.56	0.00
Do you have problems with communicating in your everyday work?	65.31	34.69	54.95	45.05	38.71	61.29	59.31	40.69	0.00

Participants’ self-assessment of their own communication skills are presented, with 1 = Good and 2 = Poor rating their own communication skills and with 1 = Agree and 2 = Disagree for all other items. Values are stated in percent (%). For each subgroup of the professions, the data of “No answer” are not displayed, but can be calculated as missing difference to 100%

In addition, 77.94% of participants (*n* = 364) rated their own communication with clients as good (Table 3). Regarding their communication in conflict situations, participants felt least confident when communicating about financial matters (61.24%; *n* = 286), dealing with emotional situations (68.31%; *n* = 319), with dominant clients (68.74%; *n* = 321), with complaints (69.81%; *n* = 326) and when communicating in stressful situations (69.81%; n = 326) (Table 4). Participants from higher age groups and those with previous professional training did not rate their own communication skills significantly better than younger participants and those who were in their first training or studies at the time of the survey. There was no significant difference between ratings of participants from different age groups or different previous professional training regarding knowledge of communication strategies (Table 4). Furthermore, 59.54% of participants (*n* = 278) answered the free-text question about which situations they found difficult when communicating with clients. In this context, the main categories “Communicating with challenging clients”, “Communicating despite own insecurities”, “Dealing with complaints” and “Discussing financial matters” could be identified. In contrast, the following main categories could be identified in the free text question about what participants found easy when communicating with clients (48.19%; *n* = 225): “Communication on a subject level”, “Communication basics”, “Friendly and respectful interaction” and “Building relationship with clients”.

**Table 3:** Participants’ self-assessment of their communication with clients

Items	Veterinary students (*n* = 294)		Trainees for veterinary nurses (*n* = 111)		Animal keeper trainees (*n* = 62)		All participants (*n* = 467)		
	1	2	1	2	1	2	1	2	No answer
How do you rate your communication with clients?	75.17	24.83	85.59	14.41	77.42	22.58	77.94	22.06	0.00
I think that I can build a stable relationship with a client.	94.90	4.76	95.50	4.50	90.32	8.06	94.43	5.14	0.43
I feel that I can win the trust of a client.	94.22	5.44	94.59	5.41	93.55	4.84	94.22	5.35	0.43
I can see how bonded a client is to his animal.	95.92	3.74	100.00	0.00	95.16	3.23	96.79	2.78	0.43
I am able to show empathy towards a client.	93.20	6.46	93.69	6.31	93.55	4.84	93.36	6.21	0.43
I think that I can conduct client meetings in a structured and logical way.	77.21	22.79	76.58	23.42	75.81	22.58	76.87	22.91	0.21
I believe that I can communicate with clients in a determined and confident way.	74.15	25.85	80.18	19.82	82.26	16.13	76.66	23.13	0.21
I would describe my way of communicating with clients as people-oriented.	77.55	22.45	84.68	14.41	77.42	20.97	79.23	20.34	0.43
I think that I can motivate clients to cooperate through appropriate communication.	84.69	15.31	83.78	15.32	85.48	12.90	84.58	14.99	0.43
I believe that I can inform clients about the services of the business.	88.10	11.90	87.39	12.61	85.48	12.90	87.58	12.21	0.21
I feel able to respond to an unexpected question from a client.	68.37	31.29	66.67	33.33	72.58	25.81	68.52	31.05	0.43
I think that I can use small talk adequately.	68.71	31.29	72.07	27.93	59.68	38.71	68.31	31.48	0.21

Participants’ self-assessment of their communication with clients are presented, with 1 = Good and 2 = Poor for the rating of the general communication with clients and with 1 = Agree and 2 = Disagree for all other items. Values are stated in percent (%). For each subgroup of the professions, the data of “No answer” are not displayed, but can be calculated as missing difference to 100%

**Table 4:** Participants’ self-assessment of their communication in conflict situations

Items	Veterinary students (*n* = 294)		Trainees for veterinary nurses (*n* = 111)		Animal keeper trainees (*n* = 62)		All participants (*n* = 467)		
	1	2	1	2	1	2	1	2	No answer
I think that I can recognise conflict situations and classify them correctly.	92.86	7.14	90.09	9.91	90.32	9.68	91.86	8.14	0.00
I know communication strategies to solve conflict situations through appropriate behavior.	46.26	53.74	72.97	27.03	50.00	50.00	53.10	46.90	0.00
I trust myself to communicate appropriately even in emotionally challenging situations in my daily work.	67.69	32.31	69.37	30.63	69.35	30.65	68.31	31.69	0.00
I feel able to make decisions in a counselling session even if I am stressed.	71.09	28.91	67.57	32.43	67.74	32.26	69.81	30.19	0.00
I think that I can communicate appropriately with an angry client.	72.11	27.21	70.27	29.73	85.48	14.52	73.45	26.12	0.43
I feel that I can deal with complaints or reminders.	68.03	31.63	68.47	31.53	80.65	19.35	69.81	29.98	0.21
I believe that I can deal with arrogant clients.	68.71	30.95	77.48	22.52	74.19	25.81	71.52	28.27	0.21
I think that I can communicate with dominant clients.	68.37	31.29	66.67	33.33	74.19	25.81	68.74	31.05	0.21
I feel able to deal with emotional clients.	79.25	20.41	81.98	18.02	83.87	16.13	80.51	19.27	0.21
I believe that I can discuss financial matters with a client.	67.69	31.97	44.14	55.86	61.29	38.71	61.24	38.54	0.21
I am convinced that I can give a client bad news about the condition of the animal.	80.27	19.39	63.96	36.04	88.71	11.29	77.52	22.27	0.21
I think that I can control my emotions during euthanasia.	87.07	12.93	92.79	6.31	83.87	16.13	88.01	11.78	0.21

Participants’ self-assessment of their communication in conflict situations are presented, with 1 = Agree and 2 = Disagree. Values are stated in percent (%). For each subgroup of the professions, the data of “No answer” are not displayed, but can be calculated as missing difference to 100%

Most participants (88.87%; *n* = 415) rated their communication level with colleagues as good or very good (Table 5). Nevertheless, 62.74% of participants (*n* = 293) answered that they did not feel sufficiently informed about the training contents of their future colleagues from other professional groups. A significantly higher proportion of veterinary students (72.11%) than of veterinary assistant trainees (41.44%) or animal keeper trainees (56.45%) did not feel sufficiently informed about the training content of their future colleagues (χ^2^: *p* < 0.0001). Regarding the free text question about what participants found difficult when communicating with colleagues (53.98%; *n* = 252), the following main categories could be identified: “Communication in case of disagreements” and “Dealing with criticism”. When asked about what participants found easy when communicating with colleagues (47.97%; *n* = 224), “Agreement of tasks”, “Mutual support” and “Communication on a subject level” could be identified as the main categories. Regarding their communication skills, 39.83% (*n* = 186) of participants felt that they were (very) poorly prepared for when starting their career (Table 5). Furthermore, 50.75% (*n* = 237) replied that their training had not helped them to improve their communication skills. Veterinary assistant trainees and animal keeper trainees felt significantly better prepared for embarking on their career with regard to their communication skills (76.58 and 77.42%) than veterinary students (50.34%) (χ^2^: *p* < 0.0001). Veterinary students denied significantly more often (62.93%) that their training had helped them to improve their communication skills than veterinary assistant trainees (29.73%) and animal keeper trainees (30.65%) (χ^2^: *p* < 0.0001). At the time of the survey, more than half of the participants (53.53%; *n* = 250) had not yet taken part in any communication training. Significantly fewer animal keeper trainees (19.35%) had received communication training than veterinary students (51.36%) and veterinary assistant trainees (40.54%) (χ^2^: *p* < 0.0001).

**Table 5:** Participants’ self-assessment of their communication within the veterinary team and of their communication skills training

Items	Veterinary students (*n* = 294)		Trainees for veterinary nurses (*n* = 111)		Animal keeper trainees (*n* = 62)		All participants (*n* = 467)		
	1	2	1	2	1	2	1	2	No answer
How do you evaluate your own communication within the veterinary team?	89.46	10.54	87.39	12.61	88.71	11.29	88.87	11.13	0.00
I feel able to represent my interests through appropriate communication within the team.	83.67	15.99	68.47	31.53	82.26	16.13	79.87	19.70	0.43
I am convinced that I can contribute to a good working atmosphere through appropriate communication within the team.	91.84	7.82	88.29	11.71	91.94	6.45	91.01	8.57	0.43
I feel able to coordinate my tasks with colleagues through appropriate communication within the team.	93.54	6.12	89.19	10.81	93.55	4.84	92.51	7.07	0.43
How well prepared do you feel for entering your profession regarding your communication skills?	50.34	49.66	76.58	23.42	77.42	22.58	60.17	39.83	0.00
Did your professional training help you to improve your communication skills?	36.73	62.93	70.27	29.73	69.35	30.65	49.04	50.75	0.21
Do you feel sufficiently informed about the training contents of your future colleagues from other professions?	27.89	72.11	58.56	41.44	43.55	56.45	37.26	62.74	0.00

Participants’ self-assessment of their communication within the veterinary team and of their communication skills training are presented, with 1 = Good and 2 = Poor for the rating of the own communication within the veterinary team and the preparation for entering the profession and with 1 = Agree and 2 = Disagree for all other items. Values are stated in percent (%). For each subgroup of the professions, the data of “No answer” are not displayed, but can be calculated as missing difference to 100%

### Participants’ attitude towards communication teaching and interprofessional education

Participants’ interest in communication skills training and interprofessional education is presented in Table 6. Significantly fewer animal keeper trainees (82.26%) found it useful to learn communication skills during their training than veterinary students (96.94%) and veterinary assistant trainees (94.59%) (χ^2^: *p* < 0.0001). Of all professional groups, veterinary students most frequently agreed that communication skills training had a positive influence on their communication with clients (Fisher’s test: *p* < 0,0001), within the veterinary team (χ^2^: *p* < 0,0001) and on their ability to deal with conflict situations (χ^2^: *p* = 0,0004) and could therefore make it easier for them to enter their profession (χ^2^: *p* < 0,0001) (Table 6). Furthermore, veterinary students were significantly more often convinced that they would feel more confident with communication in the context of euthanasia after training their communication skills (χ^2^: *p* < 0,0001). Animal keeper trainees, on the other hand, denied most frequently of all professional groups that communication skills training could lead to improved client conversations, easier entry into the profession as well as better communication in conflict situations.

**Table 6:** Participants’ interest in communication skills training and interprofessional education

Items	Veterinary students (*n* = 294)		Trainees for veterinary nurses (*n* = 111)		Animal keeper trainees (*n* = 62)		All participants (*n* = 467)		
	1	2	1	2	1	2	1	2	No answer
I believe that communication skills can be learned.	97.28	2.04	94.59	5.41	95.16	4.84	96.36	3.21	0.43
I think that it makes sense to learn communication skills during my professional training.	96.94	2.38	94.59	5.41	82.26	17.74	94.43	5.14	0.43
I think that it is appropriate to integrate communication skills training as a compulsory subject.	60.88	38.44	64.86	35.14	45.16	54.84	59.74	39.83	0.43
I would prefer to integrate communication skills training as an elective subject.	76.19	23.13	74.77	24.32	58.06	41.94	73.45	25.91	0.64
I don’t have time in my professional training to learn communication skills.	39.46	59.86	29.73	70.27	32.26	67.74	36.19	63.38	0.43
Learning communication skills improves my ability to have conversations with clients.	98.30	1.02	93.69	6.31	87.10	12.90	95.72	3.85	0.43
Learning communication skills enhances my ability to work within a team.	96.26	3.06	84.68	15.32	87.10	11.29	92.29	7.07	0.64
Teaching communication skills can make it easier for me to enter my profession.	96.60	2.72	88.29	11.71	83.87	16.13	92.93	6.64	0.43
With the help of communication training, I can learn how to deal with conflict situations in my future professional life.	93.88	5.44	85.59	14.41	80.65	19.35	90.15	9.42	0.43
I am interested in participating in an interprofessional communication training with my future colleagues.	87.07	12.24	60.36	39.64	54.84	45.16	76.45	23.13	0.43
I believe that an interprofessional communication training can improve my cooperation with my future colleagues.	95.24	3.74	86.49	13.51	75.81	24.19	90.58	8.78	0.64
An interprofessional communication training would help me to respect my future colleagues.	58.50	40.82	41.44	58.56	45.16	54.84	52.68	46.90	0.43
I would feel more confident in communicating in the context of euthanasia if I had practiced the situation beforehand.	87.76	11.90	64.86	35.14	67.74	32.26	79.66	20.13	0.21
Learning communication skills helps me to respect clients.	69.39	30.27	70.27	29.73	64.52	33.87	68.95	30.62	0.43

Participants’ interest in communication skills training and interprofessional education is presented, with 1 = Agree and 2 = Disagree. Values are stated in percent (%). For each subgroup of the professions, the data of “No answer” are not displayed, but can be calculated as missing difference to 100%

Participants in the survey showed great interest in the concept of interprofessional education (Table 6), whereas veterinary students showed significantly greater interest in interprofessional communication training (87.07%) than veterinary assistant trainees (60.36%) and animal keeper trainees (54.84%) (χ^2^: *p* < 0.0001). Furthermore, animal keeper trainees were significantly less likely to agree that an interprofessional communication training could improve their cooperation with colleagues (75.81%) than veterinary students (95.24%) and veterinary assistant trainees (86.49%) (χ^2^: *p* < 0.0001). Additionally, a significantly higher proportion of veterinary students (58.50%) than of veterinary assistant trainees (41.44%) or animal keeper trainees (45.16%) agreed that an interprofessional communication training could help them to respect their future colleagues (χ^2^: *p* = 0.0031).

## Discussion

In this study, we explored German veterinary students’, veterinary assistant trainees’ and animal keeper trainees’ perceptions of the value of communication skills. Furthermore, we tested their basic knowledge and asked them to assess their own communication skills and interest in interprofessional communication skills training.

The relevance of communication skills was ranked highly by most of the respondents, supporting research on communication as a core clinical skill in veterinary medicine [1, 3, 22, 23]. Underlining the findings of McDermott et al. [24], many veterinary students agreed that communication skills were equally important for their everyday work as clinical knowledge. This statement can be extended to the veterinary assistant trainees and the animal keeper trainees. Furthermore, the study revealed that many respondents had already experienced conflict situations in a professional context that had been triggered by a lack of communication skills. This confirms that a lack of effective communication is a frequent problem in veterinary medicine, often resulting in complaints against veterinarians [11–14] and all members of the veterinary team [10]. Furthermore, this result shows that participants were aware of their communication skills at least to the extent of recognising problems. In this study, the relevance of communication skills was ranked higher by veterinary students and veterinary assistant trainees than by animal keeper trainees. This difference between the professions may be due to different roles, assigned tasks and varying curricula. Furthermore, male participants rated communication skills as less important for their everyday professional life. This is consistent with the findings of a previous study, demonstrating that female veterinary students and graduates value professional skills more highly [25].

Even when veterinary students show a stronger theoretical knowledge, no difference can be determined regarding the self-assessment of their own communication skills between the different professional groups. Similar to the results of the survey of Meehan and Menniti, participants felt most confident when recognising how bonded a client is with his animal, building rapport, showing empathy and listening actively to a client [21]. Nevertheless, most participants admitted having problems with communication in their daily work, with veterinary students most frequently, and most expressed a demand for skills improvement. This difference could be due to the differing curricula in Germany. Consistent with findings from previous studies, respondents in this study defined situations such as discussing finances, dealing with demanding clients or communicating in emotional situations like euthanasia as difficult and challenging [21, 25, 26].

A significantly lower proportion of respondents with previous education stated that they had problems when communicating in their everyday working life. In contrast, participants from higher age groups as well as those with previous education were equally likely to state that their communication skills needed to be improved. Therefore, the factors of previous education or age have a positive influence on communication skills, but the persons concerned still need to improve these skills. Additionally, no significant difference between self-assessment of participants from different age groups or previous professional training concerning their own communication skills and their knowledge of communication strategies could be found. These findings underline that formal training during the veterinary studies or veterinary assistant and animal keeper training programmes helps to improve skills and increases confidence [14, 27]. In the worst case scenario, learning communication skills based on experience alone can even lead to reinforcing bad habits. Due to the lack of distinction between effective and poor communication, counterproductive communication can unconsciously become routine [14, 27]. This result underlines the importance of teaching effective communication skills even before entering the veterinary profession [14].

Although teaching of communication skills is currently part of the hidden curriculum in veterinary medicine in Germany, about half of the questioned participants had already taken part in communication training. Despite this fact, respondents felt poorly prepared for entering their profession and more than half of the participants responded that their training had not helped them sufficiently to improve their communication skills. These findings support previous research, showing that the majority of veterinary graduates do not feel competent in communication when completing their studies [22, 24, 28]. As a difference between the professional groups, it can be noted that veterinary students felt the least prepared by their training programmes for embarking on their professional career. This can possibly be attributed to the fact that communication theory is already an integral part of training programmes for veterinary assistants and animal keepers. Significantly fewer animal keeper trainees had already taken part in communication training compared to the other two groups. This result is surprising, as the teaching of communication skills is an integral part of animal keeper training programmes in Germany, in contrast to undergraduate veterinary medical training. At the same time, this result shows that a clear definition of the different professional groups’ role distributions and qualifications is of central importance for the implementation or adaptation of communication skills training.

The term of interprofessional communication was familiar to only 38.12% (*n* = 178) of participants and 62.74% did not feel sufficiently informed about the training contents of their future colleagues, echoing the results of previous research that interprofessional education is lacking in the veterinary medical field [5, 13]. Despite the fact that veterinary students answered significantly more often in the multiple-choice test that they were familiar with the term of interprofessional communication, a significantly higher proportion of veterinary students felt poorly informed about the training contents of their future colleagues from other professional groups.

Identified learners` great interest is consistent with findings of a previous study, demonstrating veterinary and veterinary nursing students’ interest in interprofessional education and their willingness to learn collaboratively [13]. In this survey, veterinary students showed the greatest and animal keeper trainees the least interest. This may be explained by the fact that veterinary students felt that they were inadequately informed about the training content of their future colleagues.

Due to the highly perceived value of communication skills, participants’ difficulties in different challenging situations, the pronounced wish skills improvements and the great interest in training, the findings of this study underline the need for implementation the teaching of interprofessional communication skills in veterinary medicine in Germany. The veterinary curriculum should include communication training and the curricula for veterinary assistants and animal keepers should be adapted to allow veterinary students and trainees to improve their communication skills to successfully enter their profession, to deal with challenging situations and to effectively collaborate in a veterinary team. All professions may benefit from clearer learning objectives and definitions of roles and division of responsibilities by learning together in an interprofessional training setting, which better reflects the real world. The need for implementing communication skills in the veterinary curriculum has been previously demonstrated by several studies [14, 16, 23, 25, 29–31] and should explicitly be implemented in German veterinary curricula with longitudinally increasing depth [32]. Even one-off communication training using role-playing can significantly improve students’ communication skills [33]. In order to enable optimal learning outcomes through experiential learning as well as lifelong learning, repeated training should be provided for veterinary students, trainees and recent graduates.

In particular at institutions in which training of all addressed groups is maintained, interprofessional education courses should be established. Students and trainees show great interest in participating in an interprofessional communication training with their future colleagues. Interprofessional education can potentially reduce hierarchical structures, perceived misconceptions about other professions, develop students’ awareness of the importance of effective communication in the veterinary team and reduce professional isolation [13]. Furthermore, effective interprofessional communication will have a positive influence on the clients’ satisfaction with the veterinarian services and their pet’s healthcare [31]. Although more research is needed to verify its effectiveness [6], the potential of interprofessional education is considered very high [34]. To implement an interprofessional training concept, the development of an overall curricular concept and the definition of common learning objectives and interprofessional competences are needed [18, 34]. In order to determine goals and the framework conditions in more detail, further studies are needed that are specifically dedicated to interprofessional issues in the veterinary workplace in Germany.

As a limitation of this study, it must be mentioned that the response rate for the survey could not be determined due to lack of information on the number of trainees at different schools. Moreover, while all five veterinary higher educational institutions forwarded the completed survey, it was not possible to reach all schools offering training for veterinary assistants and animal keepers. Some of the survey questions were related more to veterinarians, as other occupation-specific surveys were used as a basis for the creation. However, due to the lack of clarity about the distribution of roles in veterinary practices and the frequent confrontation of veterinary assistants and animal keepers with challenging situations in communicating with clients, these questions were deliberately sent to all professional groups. Despite these facts, the collected data provides sufficient overview to draw conclusions concerning the current status and potential future trends regarding all three professional groups. Furthermore, the evaluation of the theoretical knowledge of respondents regarding the multiple-choice test must be viewed critically. It is uncertain whether the participants used sources of information when answering the questions, that could have falsified the results. In addition, the use of self-assessment can be an inaccurate indicator of real performance [35]. Nevertheless, the results can be considered as a valuable first impression of participants’ strengths and weaknesses regarding their communication skills to identify important aspects for implementing communication skills training.

## Conclusion

This is the first study examining the interprofessional state of knowledge regarding communication skills of veterinary undergraduate students, veterinary assistant trainees and animal keeper trainees in Germany. The highly perceived relevance of communication skills by the respondents confirms the importance of communication as a core clinical skill in veterinary practice. Even if, regardless of the professional group, most students and trainees rated their own communication skills as good or very good, most participants felt that their communication skills needed to be improved and more than half admitted having problems with communication in their daily work. Although about half of the respondents had taken part in communication training, many respondents did not feel adequately prepared in communication when entering their profession. The participants did not feel sufficiently informed about the training contents of their future colleagues by other professional groups.

These findings suggest that communication training and interprofessional education concepts are inadequate in German undergraduate veterinary and veterinary-related training programmes. Indeed, the survey underlines the need for mandatory implementation of communication skills training in the veterinary medical field and adaptation of existing curricula for veterinary assistants and animal keepers in Germany.

## Data Availability

The datasets used and/or analysed during the current study are available from the corresponding author on reasonable request.
